# Realist review: understanding the challenges of medicine optimisation among older people from ethnic minority communities with polypharmacy in primary care

**DOI:** 10.1186/s12877-025-06594-1

**Published:** 2025-11-17

**Authors:** Nesrein Hamed, Clare Bates, Muhammad Umair Khan, Ian Maidment

**Affiliations:** 1 Aston University, Birmingham, England; 2 National Institute for Health and Care Research, Leeds, England; 3 University of Birmingham, Birmingham, England

**Keywords:** Multimorbidity, Person-centred care, Medication review, Medication management, Cultural competence, Deprescribing, Overprescribing, Under prescribing

## Abstract

**Introduction:**

Polypharmacy is growing among older people from ethnic minority communities in the United Kingdom, due to a higher prevalence of chronic conditions like diabetes and cardiovascular diseases. Health disparities and unequal access to healthcare services further complicate treatment. Medicine optimisation aims to identify suboptimal medication use, reduce unnecessary medications, and improve adherence. However, medicine optimisation within ethnic minority communities presents challenges because of cultural differences, language barriers, and systemic issues.

**Aims and objectives:**

To understand how Medicine optimisation works (or does not work) for older people from ethnic minority communities within primary care, identifying what works, for whom, and under what circumstances.

**Methods:**

We conducted a realist review following Pawson’s five-step framework: we developed initial programme theories, conducted systematic searches, selected and appraised studies, and synthesised the data. The review adhered to the Realist and Meta-Narrative Evidence Syntheses: Evolving Standards (RAMESES) guidelines.

**Result/discussion:**

Our programme theory is supported by 11 context-mechanism-outcome configurations that outline key contextual factors and mechanisms influencing the outcomes of Medicine optimisation. Cultural competence among practitioners is essential for building trust. Cultural norms and stigmas significantly influence how medications are perceived and adhered to, while language barriers hinder effective communication and understanding between older people and practitioners. Additionally, religious beliefs shape health behaviours and can impact adherence.

**Conclusion:**

Medicine optimisation for older people from ethnic minority communities is complex, due to the layered context in which Medicine optimisation occurs. Our review suggests that one-size-fits-all approaches to Medicine optimisation are inadequate. Significant gaps remain in understanding the contexts and mechanisms affecting Medicine optimisation in these groups, requiring further research across.

**Supplementary Information:**

The online version contains supplementary material available at 10.1186/s12877-025-06594-1.

## Introduction

 Polypharmacy is defined as the concurrent use of five medications or more by an individual in response to the associated chronic conditions [1]. Polypharmacy has become increasingly common, particularly among older people [2]. The World Health Organization (WHO) defines older age as beginning at 60 years, although many epidemiological studies use 65 years as a threshold. For this review, we adopted the WHO definition while recognising that any definition based solely on chronological age has limitations, as ageing experiences vary across groups [3]. A review estimated that about 37% of older people experience polypharmacy, with prevalence especially high in inpatient settings and associated with older age [4]. Internationally, multi-country data in European indicate a prevalence ranging from 26% to 40% among individuals aged 65 and over [5]. In the United States, polypharmacy affects as many as 65% of adults aged 65 years and older [6]. In England, one study found that about 49% of older people (aged 65 and over) were taking five or more medications [7]. Additionally, the proportion of people aged 65 and older taking five or more medications increased from 12% to 49% over the past 20 years [8]. More recently, the 2016 Health Survey for England found that 56% of individuals aged 85 and above were taking at least five medications, in comparison to 9% of individuals aged between 45 and 54 [9].

Disproportionally, polypharmacy impacts older people from ethnic minority communities (EMCs) [10]. While the rise in polypharmacy is often linked to an ageing population with a higher prevalence of multiple chronic conditions, there are other influencing factors as well. The cultural backgrounds of older people from EMCs affect health-seeking behaviours and relationships with medications [11]. They often have distinct health needs and expectations from healthcare systems compared to native populations [12]. They may seek supplementary healthcare services from their countries of origin [9]. Additionally, language barriers and the lack of support networks can worsen chronic conditions, which may lead to increased polypharmacy [9]. Socioeconomic status further complicates this issue, with higher rates of polypharmacy in more deprived areas where EMCs are more likely to reside [9, 13].

Statistics reveal significant health disparities between EMCs collectively and the general population of the United Kingdom (UK). For example, type 2 diabetes is up to three to six times more common in South Asian and Black groups compared with White populations [14]. Recent UK-based data reports that type 2 diabetes affects approximately 18% of South Asians and 13% of African or African-Caribbean people, compared with only 4% the general UK population [15]. Similarly, cardiovascular disease prevalence is higher in South Asian people, but Caribbean people have a higher risk of CVD compared to the general population [16]. These conditions contribute to higher levels of multimorbidity in EMCs, which in turn increase the likelihood of polypharmacy [17, 18].

Beyond clinical need, EMCs, particularly newer immigrant groups such as Arab communities, often face barriers such as limited English proficiency, cultural differences in health beliefs, and difficulties navigating the systems [19]. These barriers may exacerbate inappropriate prescribing, medication errors, and poor adherence, thereby increasing the risk and impact of polypharmacy in these populations [20].

Medicine optimisation (MO) is defined by the NICE guidance as “a person-centred approach to safe and effective medicines use, to ensure individuals obtain the best possible outcomes from their medicines.” [21]. Unlike medicine management, which emphasizes organizational processes, MO focuses on the individual and their outcomes [22]. MO involves several key components that can occur at any point along the patient care pathway [23]. These components include prescription ordering and delivery, prescribing, dispensing, administration, medication review, and the provision of advice and counselling [23].

The lack of effective MO can lead to immediate issues like decreased medication adherence and increased adverse drug reactions, lowering patients’ quality of life [23]. In the UK, medication-related problems are responsible for up to 6.5% of unplanned hospital admissions, costing the National Health Service (NHS) an estimated £466 million annually [24]. Long-term consequences include the progression of chronic diseases and the sustained burden of healthcare. Thus, successful MO is key to reducing the healthcare burden and enhancing the quality of care for patients, particularly among older people from EMCs, and addressing the challenges associated with polypharmacy [25].

There are numerous opportunities for practitioners based in primary care to optimise medicine use for older people from EMCs. However, these communities often face additional barriers that complicate MO, including cultural and systemic challenges [26]. Misunderstandings regarding medication use and instructions are common, and cultural or religious perceptions of medications can further affect adherence [26, 27]. In this context, culturally competent care, defined as the ability of healthcare providers to deliver care that meets the social, cultural, and language needs of patients, is an essential for MO [28]. Ensuring cultural competence can help practitioners to engage more effectively with older people from EMCs [29]. Nonetheless, factors such as limited appointment times and language differences can restrict opportunities and prevent practitioners from engaging [30].

Informal carers, typically family members or friends who provide support to older people from EMCs, play a vital role in MO for these people [31]. However, their involvement can lead to significant stress and burnout, which in turn affects the quality of care provided [32].

Overall, there is limited research in this area, including how contexts interact with mechanisms to influence the success or failure of MO for older people from EMCs. For instance, existing literature typically examine barriers to healthcare access or cultural competence without thoroughly exploring how these variables influence MO [33]​. To understand this complexity, we utilised a theory-driven approach, a realist review. A realist review aims to understand why interventions work or do not work [34]. It goes beyond identifying what works to explore how and why it works, or doesn’t, in specific circumstances [35]. This involves a more detailed examination of the interactions between context, mechanism, and outcome [35].

Although earlier work, such as the NIHR MEMORABLE realist synthesis, highlighted the complex and multi-level nature of medicines management for older people with multimorbidity, it did not fully explore the influence of cultural identity, language, and migration on MO in EMCs and recommended further research specifically focused on EMCs [36]. Our protocol paper outlined the importance of addressing this gap by focusing specifically on older people from EMCs with polypharmacy in primary care [37]. Building directly on this foundation, the present paper reports the findings of a realist review that refines and tests programme theories to explain how, why, and in what circumstances MO works or fails to work for this communities [37].

## Methods

The methods are described in detail elsewhere [37]. This review followed Pawson et al.,.‘s five-step framework and was used in other realist reviews [35, 38]. The process included developing initial programme theories (IPTs), searching for evidence, selecting, and appraising relevant studies, and synthesising the data. The approach is iterative, allowing steps to overlap for deeper insights. The review adhered to RAMESES guidelines [34] and is registered in PROSPERO (registration number CRD42023432204) [39].

In realist reviews, evidence is analysed through context–mechanism–outcome configurations (CMOCs) [40]. Context refers to the conditions in which MO takes place (e.g., social, cultural, or organisational level) [40]. Mechanism captures the underlying processes or responses triggered in those contexts (e.g., trust, stigma, communication), which explain why outcomes occur [40]. Outcomes are the intended or unintended consequences, such as adherence, satisfaction, or disengagement [41]. IPTs are tested against the evidence through these CMOCs and are iteratively refined into the final middle-range programme theory [40].

### Step 1: developing the IPTs

We began by mapping out four IPTs [hypothesised context-mechanism-outcome configurations (CMOCs)], each focusing on a different level: older people from EMCs, practitioners, informal (family) carers, and organisations [34]. We drew on: (i) empirical evidence from studies such as MEMORABLE, which highlighted challenges including workload pressures and limited patient involvement; (ii) theoretical frameworks, particularly Social Cognitive Theory (SCT), which emphasises the interaction between behaviour, personal factors, and social context; and (iii) early practitioner insights [36, 42, 43].

Practitioners were engaged in a single, yet iterative, consultation process. Draft IPTs were presented for review in one-to-one discussions, where practitioners assessed their relevance to everyday practice, highlighted gaps, and suggested refinements. Feedback was recorded, thematically summarised, and fed back into revised versions. These revisions were re-circulated to the same group until consensus was reached on up to four IPTs, which were then tested against the wider literature.

Although including a broader group, such as Patient and Public Involvement (PPI), older people from EMCs, and informal carers, would have added value, we deliberately focused solely on practitioners at this stage to ensure the IPTs captured context-specific mechanisms directly shaping MO in practice. We recognise this as a limitation, as it meant that early patient and carer perspectives were not incorporated into the initial IPT development, although these were considered in later stages of the evaluation.

Through this process, we arrived at a final set of four IPTs, which were then tested against the wider literature.

### Step 2: searching process

To refine and test the search strategy, NH first conducted pilot searches in MEDLINE and Embase. This iterative phase enabled us to trial different combinations of terms, assess their relevance, and adjust the strategy based on feedback from IM, CB, and an information scientist. Once the pilot confirmed that the strategy was both sensitive and manageable, NH conducted a comprehensive search across MEDLINE/PubMed, Embase, Scopus, Web of Science, the Cochrane Library, CINAHL, and PsycINFO from November 2023 to February 2024.

For grey literature, we searched ProQuest Dissertations & Theses Global, the King’s Fund Library Database, NHS Evidence, and NICE. To maximise coverage, backward and forward citation tracking, as well as snowballing techniques, were also employed.

The search was structured around four elements: context (older people ≥ 60 years from EMCs with polypharmacy in primary care), interventions (MO and related experiences of patients, carers, and practitioners), mechanisms (from programme theory), and outcomes (quality of life, adherence, adverse events, satisfaction, and unexpected outcomes). Appendix 1 presents the key concepts and search terms, and Appendix 2 provides the full search strategies.

### Step 3: selection and appraisal of evidence

We followed a systematic two-step process to select and appraise articles, using RAYYAN software [44].

In the first step, NH conducted an initial screening of all titles, abstracts, and keywords of potentially relevant documents, which were evaluated against the inclusion and exclusion criteria (see Table 1 for details). CB independently reviewed 20% of the screened documents. Any disagreements were resolved through discussion, or if necessary, by consulting with the rest of the team.

**Table 1:** Inclusion and exclusion criteria

Inclusion Criteria	Exclusion Criteria
*Population*: Older people aged 60 years and above from EMCs. Studies including participants younger than 60 years (18–59) were considered only if they provided transferable insights directly relevant to older people from EMCs.	Studies with no relevance to older people from EMCs or to issues of polypharmacy and MO.
*Participants*: Studies including either/or (i) older people from EMCs, (ii) their informal carers (family or friends providing support), or (iii) primary care practitioners (e.g., GPs, pharmacists, nurses) involved in their care.	Studies not involving older people, their informal carers, or practitioners relevant to MO.
*Setting*: We defined primary care broadly as first-contact, accessible, community-based health services outside hospital settings (e.g., general practice, community pharmacy).	Studies conducted exclusively in secondary, tertiary, or inpatient hospital settings.
*Focus*: Studies addressing MO interventions, broadly defined to include structured approaches (e.g., medication reviews, deprescribing), system-level or service changes (e.g., pharmacist integration), or informal strategies influencing the use of medicines.	Studies not focusing on MO interventions or that address medicine use without reference to MO.
*Designs*: Qualitative, quantitative, and mixed-methods studies; relevant grey literature (e.g., policy documents, professional guidelines, reports, opinion pieces) that provide useful insights into MO.	Studies with no clear design, low quality without usable data, or not offering relevant insights for theory development.
*Language*: English-language publications.	Non-English language publications.

For the second step, the full texts of included documents were reviewed by NH, with CB cross-checking a sample for consistency. The documents were then assessed for their relevance, rigour, and richness using a relevance ranking scale, which had been applied in previous studies [45].

All full-text articles were appraised on a 1–5-star scale for relevance, rigour, and richness, following the approach used in a previous realist review [45]:

Relevance was assessed by asking whether the article provided insights into MO for older people from EMCs with polypharmacy in primary care, either directly (older people’s perspectives) or indirectly through the views of carers or practitioners involved in their care. And whether these insights could inform or refine programme theory:

- High Relevance (4–5 Stars): studies providing direct insights into MO for older people (≥ 60 years) from EMCs in primary care, particularly in the context of polypharmacy. Studies that included participants below 60 years [18–59] were only considered if their findings were highly transferable to older people from EMCs (e.g., cultural or language-related barriers rather than age-specific biological issues).
- Moderate Relevance (3 Stars): studies that provided tangential or background information on MO but did not offer sufficient depth or direct applicability to theory development. These were excluded at the full-text stage.
- Low Relevance (2 Stars): studies discussing MO or polypharmacy without focusing on older people from EMCs, or primarily addressing significantly younger populations. Excluded.
- No Relevance (1 Star): studies not aligned with the review focus or population of interest. Excluded.

#### Rigor

This was assessed by examining the credibility of the study design, methods, and analysis. For qualitative studies, we considered sampling, reflexivity, and analytic transparency, for quantitative studies, appropriateness of design, sample size, and analysis. Mixed-methods studies were assessed for consistency and integration. While high-rigour studies were prioritised, some less rigorous studies were retained where they offered distinctive contextual or cultural insights valuable for CMOCs development (e.g., grey literature or small-scale qualitative reports).

#### Richness

This refers to the depth to which the studies examined the experiences of older people from EMCs. The studies that delved most deeply into these experiences were given greater weight and used to help refine our programme theory.

One-star and Two-star ratings indicated that the documents were irrelevant and were excluded. Three-star documents, though not directly contributing to the core theory, require further discussion with a second reviewer. Four-star and five-star documents were considered highly relevant and rich and were integrated into the analysis to directly inform and refine the programme theory.

To avoid duplication, especially from systematic reviews, each study within the systematic reviews was cross-checked against our included articles to ensure it wasn’t counted twice.

### Step 4: data extraction and analysis

We uploaded the full texts of the included papers into NVivo for data management. Key study characteristics (author, year, country, setting, study purpose, design, participant ages and numbers, ethnicity, and summary of findings) were extracted into an Excel spreadsheet for reference.

The coding process in NVivo drew on inductive, deductive, and retroductive approaches, which are well established within realist methodology [45, 46].

- Inductive coding was used to capture patterns emerging directly from the data, particularly in identifying contexts, mechanisms, and outcomes described by participants or authors.
- Deductive coding was applied, where we tested data against our IPTs, and sensitising concepts drawn from the literature.
- Retroductive reasoning was used to move beyond what was explicitly stated in the data to infer hidden causal processes, for example, theorising mechanisms (e.g., trust, cultural safety, communication barriers) that explained why particular outcomes occurred in given contexts.

This iterative process was guided by a set of realist-informed coding questions, including:

1. What are the contexts described (e.g., social, cultural, organisational) that influence MO?
2. What mechanisms are triggered in these contexts (e.g., trust, knowledge, fear, cultural beliefs)?
3. What outcomes follow from these contexts and mechanisms?
4. How does this evidence support, refine, or challenge our IPTs?

NH conducted the initial coding and developed memos to capture evolving interpretations. CB cross-checked a sample of coded transcripts and memos for consistency, with discrepancies resolved through discussion.

To deepen interpretation, we used the SCT to map personal factors (e.g., beliefs, knowledge, skills), environmental influences (e.g., cultural and social factors), and behavioural aspects (e.g., medication adherence, engagement with practitioners) onto the emerging CMOCs [43]. This facilitated a systematic connection between empirical findings and theory development. SCT did not replace the realist approach but provided a complementary lens to structure and interpret emerging CMOCs.

### Step 5: data synthesis

In this step, we employed various forms of reasoning, including juxtaposition, reconciliation, adjudication, and consolidation. Interpretive cross-case comparisons were employed to explain how and why certain outcomes occurred, examining factors such as levels of engagement in MO and cultural differences. This rigorous approach facilitated a comprehensive synthesis of the data, leading to robust and insightful conclusions [40].

Each tentative CMOC was carefully reviewed, verified against the underlying evidence, and refined. Where appropriate, similar CMOCs were merged. This iterative refinement involved rechecking data from NVivo codes, extracting relevant examples, and discussing with the project team.

## Result

### Search results

**Fig. 1:**
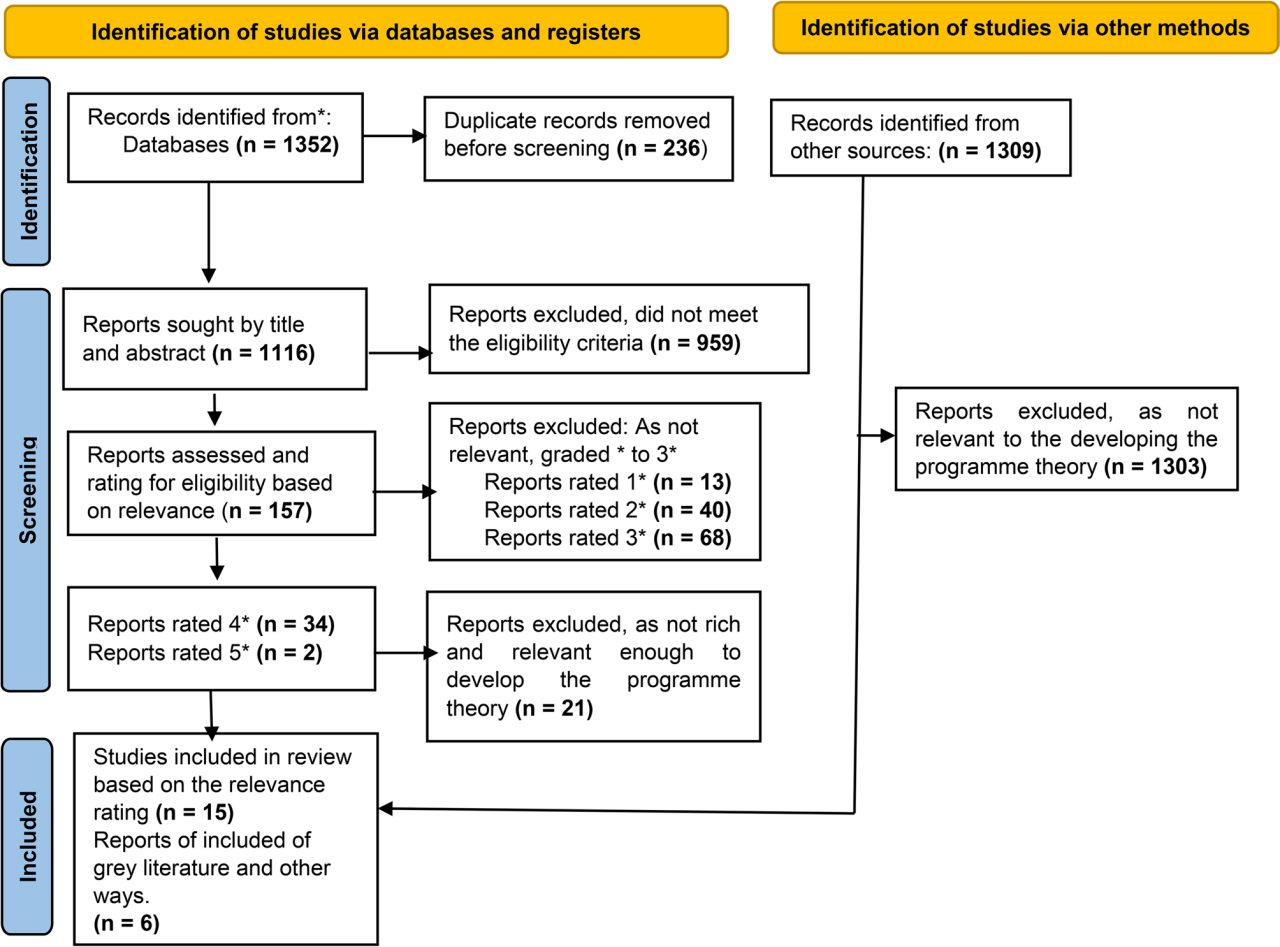
Prisma flow diagram

Our main search yielded a total of 1116 unique results. Following the initial title and abstract screening, 157 full-text documents were assessed. Of these, 2 papers received a five-star rating, while 34 papers were assigned a four-star rating. Additional sources, including citation searches, additional database searches, and personal contacts, contributed to six papers. PRISMA flow diagram for full details (Fig. 1).

After deciding to narrow the review’s focus to ensure high relevance and richness, 21 of these 36 papers were excluded. Leaving a total of 15 four-star and five-star papers identified via search and six papers via other methods. Of these, three were rated four-star and three as five-star based on their relevance and contribution to the programme theory. Details are presented in Appendix 3.

The studies varied widely in their focus, 12 papers addressed challenges in medication management within these populations, highlighting cultural misconceptions, poor health literacy, and communication barriers as major issues [47–58]. Five papers examined the impact of cultural beliefs on medication adherence [49, 56, 59–61], while four explored healthcare professionals’ perspectives on medication safety for older people from EMCs with cognitive impairments or chronic conditions [62–65]. These studies often covered overlapping themes, revealing a complex interaction between cultural, systemic, and personal factors in MO.

### The final middle-range programme theory and corresponding CMOCs

Building on the IPTs described in Step 1, we iteratively tested, refined, and consolidated CMOCs to arrive at a final middle-range programme. Table 2 presents this final middle-range programme theory alongside the corresponding CMOCs.

**Table 2:** Refined CMOCs derived from testing and consolidating IPTs against the evidence, guided by SCT

SCT component	CMOC no.	Refined CMOC	Supporting quotes and data source
**Practitioners (Knowledge, skills, beliefs about capabilities)**			
Capability	1	If Practitioners lack understanding of their older person’s cultural backgrounds (C), then it reduces trust (M), leading to non-adherence and poorer communication (O).	14 included studies supported this CMOC [47–52, 54, 56, 59, 60, 62, 63, 65, 66]. The lack of cultural competence negatively impacts the practitioners’ ability to engage older people effectively. A practitioner’s insight highlights this challenge *“If they are struggling to understand that person*,* or if they don’t understand their background*,* then they are not going to cater their treatment towards what’s best for them”* [54]. This lack of understanding often results in poor communication and mistrust. For instance, a patient’s remark, *“I let him prescribe me my medication but apart from that I never consult him”* [60], suggests that perceived cultural incompetence or indifference may contribute to reduced patient engagement and adherence.
Intention and shared decision-making	2	If the practitioners create an environment where older people from EMCs feel heard and trusted (C) then active engagement in shared decision-making will be enhanced (M), leading to greater satisfaction (O).	We identified two studies that provided support for this CMOC [51, 66]. When older people from EMCs feel heard and trusted, their engagement in shared decision-making significantly increases, which is critical for effective MO. Positive outcomes have been observed when practitioners actively involve patients in discussions about their prescribed medications, reinforcing a sense of ownership and control over their health. For instance, participants who reported being part of the decision-making process expressed greater satisfaction and a stronger commitment to adhering to their prescribed medication regimens [51]. However, this process is often challenged by systemic barriers such as the involvement of multiple specialists and poor communication between providers, which can lead to confusion and disengagement. As one participant noted, *“A central barrier to shared decision-making was having too many doctors prescribing the medications*,* too many pharmacies filling the prescriptions*,* and no sharing of information between those entities or with the patient”* [51]. Despite these challenges, those who felt supported by their practitioners were more likely to stay engaged and achieve better health outcomes, highlighting the importance of clear communication in MO.
**Patients (Health literacy, Social Influence, beliefs about consequence)**			
Cultural Stigmas	3	If cultural stigmas related to mental health are presented (C) then it could lead to avoidance of necessary medications and poorer communication with the practitioners (M), leading to issues such as unmanaged symptoms or worsening of the condition (O).	This CMOC was developed based on 11 studies [49–51, 53–55, 57, 61–64]. That emphasise how cultural norms and stigmas could influence health behaviours within these populations, particularly regarding mental health. For example, within Arab communities, there is resistance to engage in mental health treatments due to cultural perceptions of mental illnesses *“I know somebody whose doctor wants her to be on antidepressants for a different mental issue*,* and she hasn’t begun the medication. It’s hard for her to accept the fact that she needs to take the antidepressants*,* and I feel like it’s a cultural thing”* [57]. Similarly, in South-East Asian communities, there’s a chance to suppress symptoms of depression and anxiety because these conditions are associated with shame and embarrassment. As one healthcare professional noted, patients *often “suppress their symptoms of depression and anxiety due to cultural beliefs of shame and embarrassment”* [54]. Another participant added, “*Patients come with a physical problem and when you dig down into it*,* you realise that there’s mental health problems underlying it… and there’s an under-diagnosis of dementia and cognitive problems in some minority populations too*”. Moreover, the stigma is so powerful that it can lead to avoidance of medications, particularly when the practitioner is from the same ethnic background. As one participant said, *“I didn’t want him [a doctor of the same ethnicity] to have access to the list of depression medications I take… it is not something I wish my community [members] to know”* [54]. This collectively shows how cultural stigmas can lead to poor communication, non-adherence, and ultimately, ineffective MO.
Communication needs	4	If the older person experiences a language barriers and cultural differences (C) then the integration of bilingual support and culturally sensitive communication strategies (M) significantly improves their comprehension and engagement, leading to better adherence, and greater overall satisfaction (O).	Language barriers were identified in 11 included studies as being significantly obstructing healthcare engagement among EMCs [48, 49, 52–54, 56, 60–62, 64, 66]. The complexity of medical terminology and cultural context can often get lost in translation, impacting patient satisfaction and medications adherence. For instance, a provider working with South Asian patients noted, “*Sometimes the way that we describe symptoms… may not translate into the communities or other languages… it can get lost”* [54].
Information overload	5	If the older person gets overwhelmed by the medication information and a lack of personalised communication (C) then this leads to mistrust and anxiety about prescribed medications (M), resulting in poor adherence and suboptimal health outcomes (O).	9 included studies supported this CMOC [47, 48, 50, 52, 53, 56, 59, 62, 66]. Older people from EMCs often find medication information overwhelming and irrelevant, leading to non-adherence. One patient expressed, *“They said the drug information was too overwhelming*,* discouraged them from taking medicine”* [48]. The lack of personalised communication fosters mistrust, resulting in poor medication adherence and worsening health conditions.
Religious Beliefs	6	If older person view health through the lens of their religious beliefs (C) then they are less likely to engage with their practitioners about medications that conflicts with these beliefs (M), leading to suboptimal health outcomes (O).	Patients from diverse religious backgrounds interpret and manage their health through their religious beliefs, which can impact their willingness to accept medications. For example, a Muslim patient might refuse medications containing non-halal ingredients: *“The doctor focuses only on the symptoms and the suitable medicine for helping me*,* but no one focuses on the medicine*,* if it is related to certain foods*,* then this can be a big problem for Muslim culture and Muslim religion”* [64]. Moreover, another Muslim participant shared, *“Religious belief is not going to stop me taking any medications*,* actually religious [beliefs] tell you [to] take care of yourself*,* so that’s why I have to take the medicine … Yeah*,* because I mean they ask you to take care of yourself. And if you take care of yourself you will be able to take care of other people”* [59]. A Sikh participant highlighted the importance of healthcare professionals respecting and understanding different cultural and religious backgrounds: *“Sometimes (healthcare professionals) don’t understand – I told them I’m a Sikh and I’m an Indian background*,* so he knew I could have the certain medicine that maybe the Muslims can’t have with it being not Halal ingredients*,* but he didn’t understand it… to me that is a big*,* big difference… this is why it is important for us to feel that they (healthcare professionals) respect and know our cultures and our backgrounds”* [64].
Traditional Health Beliefs	7	If older person holds traditional cultural health beliefs (C), then this can significantly influence their preferences for and adherence to traditional versus contemporary medicines (M), leading to varying level of engagement with contemporary medicines.	8 studies supported that cultural and ethnic backgrounds shape patient preferences for traditional or contemporary medicine [50, 52, 56, 57, 59, 60, 64, 67]. For instance, A participant shared, *“To me*,* medicine is also a poison. So don’t depend on too much. I believe that medicine is not really good for the health. It’s good for the sickness*,* but it’s not good for the health”* [52]. This perspective shows a preference for traditional practices due to concerns about the perceived harms of Western medicines. Additionally, some patients may perceive traditional medicine as more accessible and effective due to their familiarity with these medications. For instance, one participant stated, “*Sometimes it could also be about trust if the medication is from your home country… You have faith in the place you come from*,* and now you come to a new country and their medication does not work as well”* [60].
**System and the organisation (environmental context, resources, intention)**			
Resource Constraints	8	If appointment times are too short and there are remote communication barriers (O) then the interactions between older person from EMCs and their practitioners are compromised (M), leading to miscommunication and incomplete understanding (O).	Systematic barriers as supported by 6 studies, such as limited appointment times and remote communication challenges, disproportionately affect EMCs. These barriers restrict the quality of patient-provider interactions [47, 50, 51, 54, 64, 66]. For instance, one provider noted the difficulty in accommodating patients with limited English: *“We try to book a double appointment if we have an interpreter… it’s difficult for them to access services*,* so when they do*,* then they often have lots of problems that they want to discuss…”* [54]. Older people from EMCs often require longer appointments due to language barriers and the need for cultural sensitivity in discussions. These factors require more time for effective communication and understanding, to ensure patients are fully engaged and their health needs are appropriately addressed. Also, a lack of sufficient consultation time can lead patients to rely on their own knowledge or alternative treatments due to inadequate information provided during appointments. As noted, “*due to limited consultation time*,* healthcare practitioners may be unable to provide information to patients who rely on their own knowledge around illnesses and prefer to use alternatives to medication treatments”* [47]. The need for interpreters and the challenges of remote communication further complicate interactions. One participant described the benefits of in-person consultations, emphasising that “*it’s not the same when you talk on the phone… sometimes I need to point to the box*,* want to describe my answer like that*,* or want to point to the things*,* but I cannot if it is talking on the phone… like my legs when I have the swelling in my ankles”* [64]. Additionally, limited patient-care provider interaction time makes detailed discussions harder. As reported, *“there is not enough time to talk with staff in detail” and “limited patient-care provider interaction and limited time availability are reported”* [50]. Also, participants mentioned that remote consultations, while convenient, often fell compared to in-person visits. They found it harder to pick up on nonverbal cues and discuss detailed medication information effectively. This lack of personal interaction made it challenging for healthcare providers to ensure that patients fully understood their medications, which could negatively impact medication adherence, particularly for older people from EMCs, as one participant said, *“It’s not the same when you talk on the phone… I need to point to the box*,* want to describe my answer like that*,* or want to point to the things*,* but I cannot if it is talking on the phone”* [64].
Experience-Based Adaptation	9	If the older person has previous experiences with different healthcare systems (C) then their understanding and trust may be shaped by those experiences, influencing their expectations and behaviors (M), which could lead to either enhanced or diminished engagement and adherence. (O).	As per the 5 included studies, EMCs bring diverse healthcare experiences from their home countries, influencing their perceptions and interactions with the new healthcare system [49, 54, 62, 64, 65]. For instance, factors such as how long they’ve been in the UK, whether they were born in the UK, and whether they recently arrived were discussed as barriers impacting a person’s understanding and expectations relative to their health [64]. Patients who have recently arrived might be used to different healthcare systems, such as private healthcare or none at all, depending on their country of origin [64]. For example, one participant from India, initially questioning about medication reviews, found them beneficial after understanding their purpose: *“In England*,* it is different with having the review*,* then you learn to know much more and learn how such-and-such medicine works… you can check these things every year*,* which I like better and I understand much more of the medicines now”* [64]. Moreover, healthcare professionals may not always realise that patients are unfamiliar with certain practices, such as medication reviews, from their home countries. Better explanations can help these patients understand the benefits, as one participant noted: *“They maybe explain it better for us… then we can know to understand the medicines review is existing and why it is good for us patients to have it”* [64]. Additionally, trust issues can arise when patients prefer medication from their home countries due to familiarity and perceived efficacy: *“Sometimes it could also be about trust if the medication is from your home country… You have faith in the place you come from*,* and now you come to a new country and their medication does not work as well”* [62]. Furthermore, the role of pharmacists and cost considerations in countries like Pakistan can differ significantly from the UK, influencing patients’ perceptions and interactions with the healthcare system: *“In Pakistan*,* the pharmacists – well with them you don’t even need to go to the doctor… The pharmacist has their own medicines*,* tablets*,* injections*,* everything*,* and they know how much to give of everything”* [49].
**Informal carers (Health literacy, Social Influence, beliefs about consequences)**			
Support and Engagement	10	If cultural expectations lead family members to take on caregiving responsibilities (C) then its prompt behaviours like active engagement (M). leading to improved communication and understanding (O).	Evidence from 13 studies supports this CMOC [49, 50, 53–58, 60–62, 65, 66]. In EMCs, family members often take on significant roles in patient care due to cultural expectations and language barriers. This active engagement can lead to improve the communication and the understanding. Participants frequently discussed the necessity of family members in translating during consultations. For instance, one participant noted, *“patients of South Asian or Arab backgrounds… are more likely to have more input from their children*,* which can facilitate processes like medication reconciliation”* [54]. Living with extended family provides necessary social support, helping patients navigate healthcare systems and adhere to medications. As one participant shared, *“Sometimes*,* there are too many people in the home helping*,* making it confusing at times. However*,* for most patients*,* extended family and living in a close-knit community provided needed and valued social support”* [61] Family members also act as essential Facilitators in communicating and interpreting the clinical information. This dynamic help in improving communication and understanding. As noted, *“family was identified as a key resource to all patients in coping with illness and navigating healthcare*,* from planning visits to understanding clinical information”* [65] Moreover, active family involvement helps bridge communication gaps. This is crucial, as another participant explained, *“Sometimes we experience that the relatives take responsibility for the patient and are a kind of spokesperson [for the patient]”* [62].
Caregiver Stress	11	If limited access to primary care services and reliance on informal caregiving (C), then this can lead to increased caregiver stress and emotional strain (M), resulting in caregiver burnout (O).	4 included studies supported this CMOC [54, 58, 61, 64]. Limited access to primary care services often forces older people to rely heavily on informal caregiving. This dynamic is particularly common in EMCS, where family members commonly take on significant caregiving roles. For instance, participants from South Asian and Arabs backgrounds frequently rely on their children for medication management and healthcare navigation, as highlighted by a pharmacist who noted that *“involving family members can facilitate processes like medication reconciliation”* [54] However, this reliance on family members can lead to increased stress and emotional strain. One participant shared that when relatives are responsible for translating sensitive medical information, it can be problematic and add to their stress [54]. Additionally, when caregivers are busy with work or other responsibilities, managing medical appointments and care can become overwhelming, as illustrated by a caregiver who had to accompany their non-English-speaking mother to appointments [61]. This intense involvement often leads to caregiver burnout. The emotional and physical toll of caregiving without adequate support can result in negative health outcomes for both the caregiver and the patient. For example, a participant reported significant stress due to taking on extensive caregiving duties, which eventually led to feelings of depression and burnout [58].

The final middle-range programme theory

Older people from EMCs experience multiple, interrelated barriers when it comes to managing multiple medications, due to a complex interaction between cultural beliefs, language differences, and systemic factors within primary care settings

To achieve MO for these communities, culturally tailored strategies that address these barriers, such as integrating bilingual support, providing culturally sensitive consultations are needed

For practitioners, a lack of cultural competence can reduce trust and engagement, which, if not addressed, results in poor adherence and health outcomes. Similarly, old people’ traditional health beliefs and religious values heavily influence their medication preferences, which often lead to hesitancy or resistance to certain prescribed medications.This requires practitioners to be mindful of not only the cultural contexts, but also the environmental constraints, such as limited appointment times and remote consultations

## Discussion

This realist review is the first to examine how, why, and under what circumstances MO work for older people from EMCs with polypharmacy in primary care. Unlike conventional systematic reviews, this approach focuses on understanding the contexts and mechanisms that drive its success, offering deeper insights into the complexities of MO and health outcomes. A range of study designs and publication types were included to deepen our understanding of the underlying programme theory.

Previous studies have predominantly focused on EMCs without considering age-related factors or on older populations without addressing the unique challenges faced by EMCs. Our review extends this understanding through the interplay of age, ethnicity, cultural beliefs, and migration-related changes, which significantly impact engagement with MO in primary care settings.

For example, the findings tell us the critical role of practitioners’ cultural competence in fostering trust and improving adherence across different EMCs. A lack of cultural understanding among practitioners often leads to mistrust and reduced engagement. Interestingly, ethnic matching, where older people are treated by practitioners of the same ethnic origin, did not impact their engagement with their practitioners. Instead, cultural competence was viewed as more important than shared ethnicity for successful MO. Studies show that older people respond more positively to practitioners who demonstrate cultural awareness and sensitivity, regardless of ethnic background [68]. For instance, religious beliefs may vary even within the same ethnic group, or younger practitioners may hold different perspectives than older people, which can impact engagement despite shared ethnicity.

Subsequently, once the research study was rolled out and we examined the cross-ethnicity, there was significant variability in how cultural norms and stigmas influenced the behaviours of older people in these communities. For example, mental health stigmas are particularly prevalent in many minority communities, such as Arab and South Asian communities, leading to significant underreporting and mismanagement of such conditions [69]. This cultural hesitation to engage in mental health medications results in behaviours where mental health issues are often treated as private family matters.

Language barriers were one of the key contextual influences and significantly impacted the engagement between practitioners and older people from EMCs, affecting the success of MO, with variations observed across different groups. Older people from South Asian and Arab communities often rely on family members for translation, which can help but also introduce inaccuracies. This issue is compounded by cultural norms that might prevent older people from asking questions or fully engaging with their practitioners. Study show that while interpreters can enhance understanding, they may also disrupt the personal nature of interactions, especially for older people who value direct communication [20]. In contrast, older people from East Asian backgrounds may prefer to use professional interpreters but still face challenges in fully expressing their concerns due to cultural expectations.

The perceptions of medication and illnesses among older people from EMCs vary significantly. Many Older people, particularly those from South Asian and East Asian backgrounds, find medication information overwhelming and irrelevant, leading to mistrust and anxiety about prescribed medications [70]. This results in poor adherence to medication regimens and suboptimal health outcomes. For example, some South Asian older people may not take certain health conditions seriously based on family experiences; East Asian older people may stop taking medication due to perceived side effects [71].

Also, religious beliefs play a critical role in shaping how these communities interact with their medications. For example, Muslim older people might refuse medications containing gelatine or other non-halal ingredients, impacting adherence. Similarly, Sikh older people emphasise the importance of recognising and respecting dietary restrictions that differ from those of Muslims. In contrast, some older people from African Caribbean Christian groups may view illness as part of a divine plan, which can lead to prioritising spiritual healing over contemporary medications [72, 73].

Variations among older people from different cultural and ethnic backgrounds extend to the use of traditional and herbal medications, which are often deeply rooted in cultural beliefs and practices. For instance, Malaysian Chinese older people express a strong preference for traditional Chinese medicines over contemporary options due to cultural familiarity and ancestral beliefs [54]. Similarly, Arab older people often incorporate natural remedies, such as cinnamon, into their diabetes management, influenced by cultural practices and advice from within their community [56]. South Asian older people frequently use Ayurveda and other herbal treatments, viewing them as less harmful and more aligned with natural healing philosophies [50]. These practices can create significant challenges in achieving MO, highlighting the need for practitioners to respect and integrate these diverse health beliefs into MO [60, 67].

Family involvement is crucial in MO among older people from various EMCs, often due to cultural norms and language barriers. This pattern is observed across different groups, including Turkish, South Asian, and other EMCs, where strong family support plays a vital role in managing complex medication regimens. However, this involvement can lead to significant stress and burnout for informal carers, who are frequently responsible for translating medical information and ensuring medication adherence [54, 58]. Additionally, while multigenerational households can provide essential support, they may also create confusion when too many family members are involved in healthcare decisions, a challenge likely prevalent across many EMCs, though specific evidence may vary.

The systemic context in primary care, including limited appointment times and challenges with remote communication, significantly impacts interactions with these populations. These barriers restrict the quality of interactions between older people and practitioners, leading to low satisfaction [20]. For instance, participants noted that remote consultations lack the benefits of in-person interactions, making it difficult to communicate nonverbal cues and detailed information about medications [74]. Limited appointment times prevent thorough discussions, especially when interpreters are needed.

The literature suggests that older people from EMCs, particularly those with limited exposure to regulated healthcare systems, often find concepts such as regular medication reviews unfamiliar and initially unnecessary [75]. This is particularly clear among South Asian communities, including those from Pakistan, who may perceive differences in the roles of pharmacists and the cost of medications compared to their countries of origin. These experiences contribute to a lower level of trust and engagement with UK primary care settings.

Moreover, the level of integration, including the length of stay in the host country, significantly influences these interactions [76]. Older people who have been in the UK for longer tend to have better integration into the healthcare system, potentially leading to higher levels of trust and engagement. However, newer immigrants might struggle more with understanding and adapting to the UK’s primary care system’s expectations, which could exacerbate their sense of disconnection and lead to lower adherence to prescribed medications.

### Limitations

The review has some limitations like any other review. Firstly, the search strategy may have missed some relevant studies, particularly those in languages other than English or not indexed in the selected databases. This could limit the generalisability of the findings.

Secondly, the review aimed to identify key contexts and mechanisms rather than provide a detailed account of all relationships between CMOCs, meaning it does not conclusively establish the effectiveness of the identified mechanisms. Further research is needed to test and refine these theories.

Lastly, there was relatively little literature available on older people from African Caribbean and mixed-race communities, reflecting a gap in the available literature. This may affect the applicability of findings to these communities. Addressing this gap in future research could enhance the inclusivity and generalisability of MO across EMCs.

### Future research

This review is part of an ongoing project that will further test the CMOCs through qualitative interviews (Realist Evaluation). This will offer practical insights into the contexts and mechanisms that shape MO outcomes in these communities. This approach aims to inform the development of more culturally sensitive and effective interventions.

## Conclusion

This review highlights the complexity of MO for older people from EMCs due to the layered context in which MO occurs. The interaction between older people and practitioners is influenced by cultural norms, beliefs, and language barriers, which shape the mechanisms that underpin successful MO. For example, the cultural background of older people can influence their health beliefs, potentially leading to different expectations or preferences for medications. On the practitioners’ side, the ability to recognise and respond to these contexts is crucial in activating positive mechanisms, such as building trust.

However, there are still significant gaps in understanding the full range of contexts and mechanisms that influence MO in these populations. Further research is needed to evaluate these factors across EMCs.

## Supplementary Information

Supplementary Material 1.

Supplementary Material 2.

Supplementary Material 3.

## Data Availability

All data generated or analysed during this study are included in this published article and its supplementary information files. The data extraction matrix and coding framework are available from the corresponding author on reasonable request.

## Abbreviations

CMOCs: Context, Mechanism, Outcome Configurations
EMCs: Ethnic Minority Communities
IPTs: The Initial Programme Theories
MO: Medicine Optimisation
NHS: National Health Service
PPI: Patient and Public Involvement
RAMESES: Realist and Meta-narrative Evidence Syntheses: Evolving Standards
SCT: Social Cognitive Theory
UK: United Kingdom
WHO: World health organisation
