# Supplementary material for: Realist review: understanding the challenges of medicine optimisation among older people from ethnic minority communities with polypharmacy in primary care
Source: BMC Geriatr. 2025 Nov 17;25:914. doi: 10.1186/s12877-025-06594-1 (PMC12625021; doi:10.1186/s12877-025-06594-1)
Supplement: Supplementary file 1 — Supplementary Material 1. [file 12877_2025_6594_MOESM1_ESM.docx]

**Appendix 1** present key concepts and associated search terms.

| Concept | Search Threads (Keywords, Synonyms, & Related Terms) |
| --- | --- |
| **1. Medicines Optimisation** | Medic* Management OR medic* optimi?ation OR drug utili?aton review OR medic* reconcil* OR medic* review OR structured medic* review OR deprescri* |
| **2. Outcome(s)** | Medic* adherence OR Medic* compliance OR patient satisfaction OR “Inappropriate prescribing” OR Overprescrib* OR “Medication burden” OR “adverse events” |
| **3. Older People (60+ years)** | older OR elderly OR aging OR "old age" OR "late life" OR "60 years and above” OR Geriatric* |
| **4. Minority Ethnic Communities** | Middle East* OR Afric* OR Asia* OR Caribbean OR "West Indies" OR Bangladesh* OR China* OR India* OR Somali* OR Ethiopia* OR Nigeria* OR Kenya* OR Uganda OR Syria* OR Pakistan* OR ethnic minorit* OR Black OR Asian OR "people of colour" OR Race* OR "mixed race" OR "mixed racial" OR "Black British" OR "indian subcontinent" OR Gyps* OR "irish traveller" OR "African Americans" OR "Asian Americans" OR Blacks OR "Hispanic Americans" OR Arabi* OR Hindu* OR Hindi OR Muslim OR Islam* OR Tamil* OR Lanka* OR Urdu OR Bengali* OR Emigran* OR Immigran* OR Refugee* OR migrant* OR asylum seeker* OR BAME OR BME |
| **5. Polypharmacy** | Polypharmacy OR multipl* medic* OR multipl* drug* OR many medic* OR “many drugs” |
| **6. Primary Care** | “Primary care” OR “Community health” OR Family practi?e OR General Practitioner OR Pharmacy OR GP OR Family Medicine |
