# Supplementary material for: Realist review: understanding the challenges of medicine optimisation among older people from ethnic minority communities with polypharmacy in primary care
Source: BMC Geriatr. 2025 Nov 17;25:914. doi: 10.1186/s12877-025-06594-1 (PMC12625021; doi:10.1186/s12877-025-06594-1)
Supplement: Supplementary file 2 — Supplementary Material 2. [file 12877_2025_6594_MOESM2_ESM.docx]

**Appendix 2:** main databases that were searched.
**Embase**

Host: Ovid

Date searched: 23/11/2023.

Searcher: NH

Hits n = 269

| 1 | (Medic* Management or medic* optimi?ation or drug utili?aton review or medic* reconcil* or medic* review or structured medic* review or deprescri*).m_titl. | 12406 |
| --- | --- | --- |
| 2 | (Medic* adherence or Medic* compliance or patient satisfaction or "Inappropriate prescribing" or Overprescrib* or "Medication burden" or "adverse events").m_titl. | 40928 |
| 3 | (older or elderly or aging or "old age" or "late life" or "60 years and above" or Geriatric*).m_kw, ab, titl. | 1976798 |
| 4 | (Middle East* or Afric* or Asia* or Caribbean or "West Indies" or Bangladesh* or China* or India* or Somali* or Ethiopia* or Nigeria* or Kenya* or Uganda or Syria* or Pakistan* or ethnic minorit* or Black or Asian or "people of colour" or Race* or "mixed race" or "mixed racial" or "Black British" or "indian subcontinent" or Gyps* or "irish traveller" or "African Americans" or "Asian Americans" or Blacks or "Hispanic Americans" or Arabi* or Hindu* or Hindi or Muslim or Islam* or Tamil* or Lanka* or Urdu or Bengali* or Emigran* or Immigran* or Refugee* or migrant* or asylum seeker* or BAME or BME). m_kw, ab, titl. | 2529523 |
| 5 | (Polypharmacy or multipl* medic* or multipl* drug* or many medic* or "many drugs"). m_kw, ab, titl. | 75407 |
| 6 | ("Primary care" or "Community health" or Family practi?e or General Practitioner or Pharmacy or GP or Family Medicine). m_kw, ab, titl. | 609399 |
| 7 | 1 or 2 or 5 | 126360 |
| 8 | 3 and 4 and 6 and 7 | 269 |

**Web of science**

Host: Web of Science (Clarivate Analytics)

Date searched: 26/11/2023.

Searcher: NH

Hits n = 599

| 1 | TI=(Medic* Management OR medic* optimi?ation OR drug utili?aton review OR medic* reconcil* OR medic* review OR structured medic* review OR deprescri* ) and Preprint Citation Index (Exclude – Database) | 66,285 |
| --- | --- | --- |
| 2 | TI=(Medic* adherence OR Medic* compliance OR patient satisfaction OR “Inappropriate prescribing” OR Overprescrib* OR “Medication burden” OR “adverse events”) and Preprint Citation Index (Exclude – Database) | 62,013 |
| 3 | TS=(older OR elderly OR aging OR "old age" OR "late life" OR "60 years and above” OR Geriatric*) and Preprint Citation Index (Exclude – Database) | 13,267,587 |
| 4 | TS=(Middle East* OR Afric* OR Asia* OR Caribbean OR "West Indies" OR Bangladesh* OR China* OR India* OR Somali* OR Ethiopia* OR Nigeria* OR Kenya* OR Uganda OR Syria* OR Pakistan* OR ethnic minorit* OR Black OR Asian OR "people of colour" OR Race* OR "mixed race" OR "mixed racial" OR "Black British" OR "indian subcontinent" OR Gyps* OR "irish traveller" OR "African Americans" OR "Asian Americans" OR Blacks OR "Hispanic Americans" OR Arabi* OR Hindu* OR Hindi OR Muslim OR Islam* OR Tamil* OR Lanka* OR Urdu OR Bengali* OR Emigran* OR Immigran* OR Refugee* OR migrant* OR asylum seeker* OR BAME OR BME ) and Preprint Citation Index (Exclude – Database) | 1,626,733 |
| 5 | TS=(Polypharmacy OR multipl* medic* OR multipl* drug* OR many medic* OR “many drugs” ) and Preprint Citation Index (Exclude – Database) | 5,501,098 |
| 6 | (TS=(“Primary care” OR “Community health” OR Family practi?e OR General Practitioner OR Pharmacy OR GP OR Family Medicine )) NOT (SILOID==("PPRN")) | 1,281,132 |
| 7 | #1 OR #2 and Preprint Citation Index (Exclude – Database) | 126,566 |
| 8 | #3 AND #4 AND #5 AND #6 AND #7 and Preprint Citation Index (Exclude – Database) | 599 |

**PsycINFO**

Host: ProQuest

Date searched: 09/12/2023.

Searcher: NH

Hits n = 26

| S1 | title(Medic* adherence OR Medic* compliance OR patient satisfaction OR “Inappropriate prescribing” OR Overprescrib* OR “Medication burden” OR “adverse events”) | 6,180 |
| --- | --- | --- |
| S2 | title(Medic* Management OR medic* optimi?ation OR drug utili?aton review OR medic* reconcil* OR medic* review OR structured medic* review OR deprescri*) | 4,948 |
| S3 | summary(older OR elderly OR aging OR "old age" OR "late life" OR "60 years and above” OR Geriatric*) | 274,223 |
| S4 | summary(Middle East* OR Afric* OR Asia* OR Caribbean OR "West Indies" OR Bangladesh* OR China* OR India* OR Somali* OR Ethiopia* OR Nigeria* OR Kenya* OR Uganda OR Syria* OR Pakistan* OR ethnic minorit* OR Black OR Asian OR "people of colour" OR Race* OR "mixed race" OR "mixed racial" OR "Black British" OR "indian subcontinent" OR Gyps* OR "irish traveller" OR "African Americans" OR "Asian Americans" OR Blacks OR "Hispanic Americans" OR Arabi* OR Hindu* OR Hindi OR Muslim OR Islam* OR Tamil* OR Lanka* OR Urdu OR Bengali* OR Emigran* OR Immigran* OR Refugee* OR migrant* OR asylum seeker* OR BAME OR BME ) | 380,480 |
| S5 | title(Polypharmacy OR multipl* medic* OR multipl* drug* OR many medic* OR “many drugs” ) | 1,673 |
| S6 | summary(“Primary care” OR “Community health” OR Family practi?e OR General Practitioner OR Pharmacy OR GP OR Family Medicine ) | 107,746 |
| S7 | [S1] OR [S2] OR [S5] | 12,394 |
| S8 | [S3] AND [S4] AND [S6] AND [S7] | 26 |

**MEDLINE**

Host: Ovid

Date searched: 02/12/2023.

Searcher: NH

Hits n = 115

| 1 | (Medic* Management or medic* optimi?ation or drug utili?aton review or medic* reconcil* or medic* review or structured medic* review or deprescri*).m_titl. | 8547 |
| --- | --- | --- |
| 2 | (Medic* adherence or Medic* compliance or patient satisfaction or "Inappropriate prescribing" or Overprescrib* or "Medication burden" or "adverse events").m_titl. | 27154 |
| 3 | (older or elderly or aging or "old age" or "late life" or "60 years and above" or Geriatric*).m_kw, ab, titl. | 1197152 |
| 4 | (Middle East* or Afric* or Asia* or Caribbean or "West Indies" or Bangladesh* or China* or India* or Somali* or Ethiopia* or Nigeria* or Kenya* or Uganda or Syria* or Pakistan* or ethnic minorit* or Black or Asian or "people of colour" or Race* or "mixed race" or "mixed racial" or "Black British" or "indian subcontinent" or Gyps* or "irish traveller" or "African Americans" or "Asian Americans" or Blacks or "Hispanic Americans" or Arabi* or Hindu* or Hindi or Muslim or Islam* or Tamil* or Lanka* or Urdu or Bengali* or Emigran* or Immigran* or Refugee* or migrant* or asylum seeker* or BAME or BME). m_kw, ab, titl. | 1927533 |
| 5 | (Polypharmacy or multipl* medic* or multipl* drug* or many medic* or "many drugs"). m_kw, ab, titl. | 5020 |
| 6 | ("Primary care" or "Community health" or Family practi?e or General Practitioner or Pharmacy or GP or Family Medicine). m_kw, ab, titl. | 426265 |
| 7 | 1 or 2 or 5 | 40532 |
| 8 | 3 and 4 and 6 and 7 | 115 |

**Cochrane library**

Host: Cochrane Library

Date searched: 29/11/2023.

Searcher: NH

Hits n = 231

| #1 | (Medic* Management or medic* optimi?ation or drug utili?aton review or medic* reconcil* or medic* review or structured medic* review or deprescri*):ti | 2793 |
| --- | --- | --- |
| #2 | (Medic* adherence or Medic* compliance or patient satisfaction or "Inappropriate prescribing" or Overprescrib* or "Medication burden" or "adverse events"):ti | 6018 |
| #3 | (older or elderly or aging or "old age" or "late life" or "60 years and above" or Geriatric*):ti,ab,kw | 182508 |
| #4 | (Middle East* or Afric* or Asia* or Caribbean or "West Indies" or Bangladesh* or China* or India* or Somali* or Ethiopia* or Nigeria* or Kenya* or Uganda or Syria* or Pakistan* or ethnic minorit* or Black or Asian or "people of colour" or Race* or "mixed race" or "mixed racial" or "Black British" or "indian subcontinent" or Gyps* or "irish traveller" or "African Americans" or "Asian Americans" or Blacks or "Hispanic Americans" or Arabi* or Hindu* or Hindi or Muslim or Islam* or Tamil* or Lanka* or Urdu or Bengali* or Emigran* or Immigran* or Refugee* or migrant* or asylum seeker* or BAME or BME):ti,ab,kw | 111061 |
| #5 | (Polypharmacy or multipl* medic* or multipl* drug* or many medic* or "many drugs"):ti,ab,kw | 82674 |
| #6 | ("Primary care" or "Community health" or Family practi?e or General Practitioner or Pharmacy or GP or Family Medicine):ti,ab,kw | 49764 |
| #7 | #1 or #2 or# 5 | 90351 |
| #8 | #3 and #4 and #6 and #7 | 231 |

**Scopus**

Host: Scopus.com

Date searched: 02/12/2023.

Searcher: NH

Hits n = 0

| **( TITLE-ABS-KEY ( medic* AND management OR medic* AND optimi?ation OR drug AND utili?aton AND review OR medic* AND reconcil* OR medic* AND review OR structured AND medic* AND review OR deprescri* ) ) AND ( TITLE-ABS-KEY ( medic* AND adherence OR medic* AND compliance OR patient AND satisfaction OR "Inappropriate prescribing" OR overprescrib* OR "Medication burden" OR "adverse events" ) ) AND ( TITLE-ABS-KEY ( older OR elderly OR aging OR "old age" OR "late life" OR "60 years and above" OR geriatric* ) ) AND ( TITLE-ABS-KEY ( middle AND east* OR afric* OR asia* OR caribbean OR "West Indies" OR bangladesh* OR china* OR india* OR somali* OR ethiopia* OR nigeria* OR kenya* OR uganda OR syria* OR pakistan* OR ethnic AND minorit* OR black OR asian OR "people off colour" OR race* OR "mixed race" OR "mixed racial" OR "Black British" OR "indian subcontinent" OR gyps* OR "irish traveller" OR "African Americans" OR "Asian Americans" OR blacks OR "Hispanic Americans" OR arabi* OR hindu* OR hindi OR muslim OR islam* OR tamil* OR lanka* OR urdu OR bengali* OR emigran* OR immigran* OR refugee* OR migrant* OR asylum AND seeker* OR bame OR bme ) ) AND ( TITLE-ABS-KEY ( polypharmacy OR multipl* AND medic* OR multipl* AND drug* OR many AND medic* OR "many drugs" ) ) AND ( TITLE-ABS-KEY ( "Primary care" OR "Community health" OR family AND practi?e OR general AND practitioner OR pharmacy OR gap OR family AND medicine ) )** |
| --- |

**CINAHL**

Host: EbscoHOST

Date searched: 05/12/2023.

Searcher: NH

Hits n = 64

| **S8** | ((AB “Primary care” OR “Community health” OR Family practi?e OR General Practitioner OR Pharmacy OR GP OR Family Medicine) AND (S1 OR S2 OR S5)) AND (S3 AND S4 AND S6 AND S7) | 64 |
| --- | --- | --- |
| **S7** | (AB “Primary care” OR “Community health” OR Family practi?e OR General Practitioner OR Pharmacy OR GP OR Family Medicine) AND (S1 OR S2 OR S5) | 4,822 |
| **S6** | AB “Primary care” OR “Community health” OR Family practi?e OR General Practitioner OR Pharmacy OR GP OR Family Medicine | 166,103 |
| **S5** | TI Polypharmacy OR multipl* medic* OR multipl* drug* OR many medic* OR “many drugs | 4,361 |
| **S4** | AB Middle East* OR Afric* OR Asia* OR Caribbean OR "West Indies" OR Bangladesh* OR China* OR India* OR Somali* OR Ethiopia* OR Nigeria* OR Kenya* OR Uganda OR Syria* OR Pakistan* OR ethnic minorit* OR Black OR Asian OR "people of colour" OR Race* OR "mixed race" OR "mixed racial" OR "Black British" OR "indian subcontinent" OR Gyps* OR "irish traveller" OR "African Americans" OR "Asian Americans" OR Blacks OR "Hispanic Americans" OR Arabi* OR Hindu* OR Hindi OR Muslim OR Islam* OR Tamil* OR Lanka [...](javascript:showHistoryTerm('ctl00_ctl00_FindField_FindField_historyControl_HistoryRepeater_ctl04_ellipsis',true)) | 329,065 |
| **S3** | AB older OR elderly OR aging OR "old age" OR "late life" OR "60 years and above” OR Geriatric* | 317,593 |
| **S2** | TI Medic* adherence OR Medic* compliance OR patient satisfaction OR “Inappropriate prescribing” OR Overprescrib* OR “Medication burden” OR “adverse events” | 21,749 |
| **S1** | TI Medic* Management OR medic* optimi?ation OR drug utili?aton review OR medic* reconcil* OR medic* review OR structured medic* review OR deprescri | 21,617 |

**PubMed**

Host: NCBI

Date searched: 30/11/2023.

Searcher: NH

Hits n = 48

| #1 | Medic* Management[Title/Abstract] OR medic* optimi?ation[Title/Abstract] OR drug utili?aton review[Title/Abstract] OR medic* reconcil*[Title/Abstract] OR medic* review[Title/Abstract] OR structured medic* review[Title/Abstract] OR deprescri*[Title/Abstract] | 1,881,851 |
| --- | --- | --- |
| #2 | Medic* adherence[Title/Abstract] OR Medic* compliance[Title/Abstract] OR patient satisfaction[Title/Abstract] OR "Inappropriate prescribing"[Title/Abstract] OR Overprescrib*[Title/Abstract] OR "Medication burden"[Title/Abstract] OR "adverse events"[Title/Abstract] | 440,074 |
| #3 | older[Title/Abstract] OR elderly[Title/Abstract] OR aging[Title/Abstract] OR "old age"[Title/Abstract] OR "late life"[Title/Abstract] OR "60 years and above"[Title/Abstract] OR Geriatric*[Title/Abstract] | 1,059,114 |
| #4 | Middle East*[Title/Abstract] OR Afric*[Title/Abstract] OR Asia*[Title/Abstract] OR Caribbean[Title/Abstract] OR "West Indies"[Title/Abstract] OR Bangladesh*[Title/Abstract] OR China*[Title/Abstract] OR India*[Title/Abstract] OR Somali*[Title/Abstract] OR Ethiopia*[Title/Abstract] OR Nigeria*[Title/Abstract] OR Kenya*[Title/Abstract] OR Uganda[Title/Abstract] OR Syria*[Title/Abstract] OR Pakistan*[Title/Abstract] OR ethnic minorit*[Title/Abstract] OR Black[Title/Abstract] OR Asian[Title/Abstract] OR "people of colour"[Title/Abstract] OR Race*[Title/Abstract] OR "mixed race"[Title/Abstract] OR "mixed racial"[Title/Abstract] OR "Black British"[Title/Abstract] OR "indian subcontinent"[Title/Abstract] OR Gyps*[Title/Abstract] OR "irish traveller"[Title/Abstract] OR "African Americans"[Title/Abstract] OR "Asian Americans"[Title/Abstract] OR Blacks[Title/Abstract] OR "Hispanic Americans"[Title/Abstract] OR Arabi*[Title/Abstract] OR Hindu*[Title/Abstract] OR Hindi[Title/Abstract] OR Muslim[Title/Abstract] OR Islam*[Title/Abstract] OR Tamil*[Title/Abstract] OR Lanka*[Title/Abstract] OR Urdu[Title/Abstract] OR Bengali*[Title/Abstract] OR Emigran*[Title/Abstract] OR Immigran*[Title/Abstract] OR Refugee*[Title/Abstract] OR migrant*[Title/Abstract] OR asylum seeker*[Title/Abstract] OR BAME[Title/Abstract] OR BME[Title/Abstract] | 1,645,601 |
| #5 | Polypharmacy[Title/Abstract] OR multipl* medic*[Title/Abstract] OR multipl* drug*[Title/Abstract] OR many medic*[Title/Abstract] OR "many drugs"[Title/Abstract] | 342,807 |
| #6 | "Primary care" OR "Community health" OR Family practi?e OR General Practitioner OR Pharmacy OR GP OR Family Medicine | 1,651,014 |
| #7 | #1 and #2 and #3 and #4 and #5 and #6 | 48 |
