# Supplementary material for: Realist review: understanding the challenges of medicine optimisation among older people from ethnic minority communities with polypharmacy in primary care
Source: BMC Geriatr. 2025 Nov 17;25:914. doi: 10.1186/s12877-025-06594-1 (PMC12625021; doi:10.1186/s12877-025-06594-1)
Supplement: Supplementary file 3 — Supplementary Material 3. [file 12877_2025_6594_MOESM3_ESM.docx]

**Appendix 3:** present the characteristics of the included studies.

| Author, year | Setting,  country | purpose Study purpose | Manuscript design | Participants (ages and numbers) | Participants’ ethnicity | Summary of findings | Relevance Ranking |
| --- | --- | --- | --- | --- | --- | --- | --- |
| Alhomoud *et al.,*  2015. | Pharmacies in London, UK | To describe medicine-related problems experienced by South Asian and Middle Eastern patients, focusing on cultural factors. | Qualitative study with semi-structured interviews. | 80 participants, (range not specified). | South Asian and Middle Eastern. | The study found that cultural factors significantly impacted how these patients managed their medications. For example, religious practices like fasting during Ramadan led to adjustments in medication routines, and cultural beliefs influenced the widespread use of non-prescription medicines. Many patients heavily relied on family members to make healthcare decisions, and frequent travel back to their home countries or for religious purposes added complexity to their medication management. The study also identified barriers such as illiteracy, language and communication difficulties, the lack of translated medical resources, and gender preferences in healthcare interactions, all of which further complicated their ability to effectively manage their medications. | The article was identified via citation search; 5*. |
| Christopher *et al.,* 2023. | Primary settings in Penang, Malaysia. | To assess medication use challenges among older people and explore contributing factors. | Mixed-methods study; cross-sectional survey and semi-structured interviews. | 393 participants for the survey, 15 for interviews, aged 60 and above. | Predominantly Chinese, followed by Malay, Indian. | The study revealed that older people commonly experienced problems like taking multiple medications (polypharmacy), difficulties sticking to their prescribed routines, and a lack of understanding about the potential side effects of their medicines. Although around 55% of participants were generally satisfied with the healthcare services they received, the findings indicated that there is still significant room for improvement, particularly in areas like patient education and better management of medications. Key factors such as forgetfulness, language barriers, and transportation difficulties were found to have a major impact on how well older people managed their medications. | 4* |
| Fadil *et al.,* 2020. | Community settings, United states. | To explore the impact of health literacy and perceived discrimination on healthcare use and medication adherence among Arab-descent Americans. | Mixed-methods study; qualitative phase with focus groups and quantitative phase with cross-sectional online survey | 27 participants for qualitative phase, 210 for quantitative phase, ages range not specified. | Arab-descent Americans. | This dissertation found that how well people understand health information and their experiences with discrimination can shape how they use healthcare and follow their treatment plans. factors income, and cultural beliefs also played big roles in whether they engaged with healthcare services. This study highlights the importance of addressing cultural sensitivities and the impact of discrimination in healthcare for Arab-descent communities. | 4* |
| Greenwood *et al.,* 2014. | London, United Kingdom. | To identify ethnic minority carers' perceptions of barriers to accessing social care and their satisfaction with these services. | Systematic review of qualitative and quantitative literature. | 13 studies included, participant ages and numbers vary. | Minority ethnic carers, including Black African, Black Caribbean, South Asian (Indian, Pakistani, Bangladeshi), and others. | The review identified significant barriers faced by carers from EMCs in accessing social care services, such as language barriers and cultural appropriateness. It also highlighted a lack of research on carers' satisfaction and the need for culturally sensitive service provision. Barriers common to all carers included hesitation to use services and low awareness of available support. While the study did not specifically mention issues related to medication use, it highlighted significant barriers that could indirectly affect access to and satisfaction with health-related services, including medication. | The article was identified via citation search; 4*. |
| Hagiwara *et al.,* 2013. | Primary care clinic, Midwestern, United states. | To examine the impact of racial bias in non-Black physicians and perceived discrimination by Black patients on communication and treatment adherence. | Secondary analysis of self-report and video-recorded data. | 112 Black patients (age range 18-65), and 14 non-Black physicians. | Black patients, non-Black physicians. | The study observed that non-Black physicians, especially those with higher implicit racial biases, tended to speak more during medical interactions, overshadowing their Black patients. In contrast, Black patients who reported experiencing more discrimination in the past spoke less during their appointments. Notably, the balance of talk time between physician and patient had a direct impact on treatment adherence: interactions where patients spoke more than usual resulted in poorer adherence to the prescribed medical treatments over time. This suggests that both physician and patient racial attitudes significantly affect communication dynamics and subsequent health behaviour outcomes in racially discordant medical interactions. | 4* |
| Jamil *et al.,* 2022. | Pittsburgh, United states. | To explore how cultural perspectives of South Asian communities affect medication adherence for type 2 diabetes and cardiovascular disease. | Qualitative research with semi-structured interviews. | 12 participants, ages range between 49 and 75. | South Asian communities (Predominantly Pakistani, followed by Indian and Bangladeshi). | The study explores how cultural beliefs and behaviours of South Asian communities impact their adherence to diabetes medications. Five key themes were identified, including the role of numerical health results in motivating care, the importance of open communication, value of self-management and autonomy, influence of religious beliefs on medication adherence, and the use of complementary and alternative medicines alongside contemporary medications. | 4* |
| Knowles *et al.,* 2016. | Manchester, United Kingdom | To explore how carers of people with common long-term conditions understand their caregiving role and seek support. | Qualitative study using semi-structured interviews. | 19 informal carers (ages not specified). | Mixed (White British, Pakistani, Indian, Black British) . | This study explores how people who care for those with long-term conditions often don't see themselves as official carers, which affects their ability to get the support they need. Many provide emotional support more than physical care, which doesn't fit the usual idea of caregiving. For EMCs, cultural expectations and a strong sense of family duty often blur the lines of caregiving, and language barriers make it even harder to find help. The study emphasises the need for better recognition of these "hidden carers" to ensure they receive adequate support. | The article was identified via citation search; 4*. |
| Lynnerup *et al.,* 2022. | Denmark | To explore healthcare professionals' perspectives on medication safety among older people from EMCs with cognitive impairment. | Qualitative study with focus groups and one semi-structured interview. | 34 healthcare practitioners (including GP, Pharmacists, Pharmacy Technicians, Nurses, and Social Workers) | Not specified. | This study investigates health care professionals' perspectives on medication safety for older people from EMCs with polypharmacy. Key themes identified were the importance of relationships in medication safety, cultural and financial barriers, and systemic issues within the health care system. Challenges included language barriers, reliance on family members for interpretation, and issues with medication management systems. The study highlights the need for improved collaboration and communication strategies across healthcare sectors to enhance medication safety for this vulnerable population. | 4* |
| Mishra *et al.,* 2011 | Family medicine practice, Baltimore, United States | To explore facilitators and barriers to medication adherence among low-income patients with chronic physical and mental health conditions. | Qualitative study using focus groups. | 50 participants, ages 40 and above. | Predominantly African American, with some White participants. | This study explored barriers and facilitators to medication adherence among participants. Barriers included medication side effects, fear of dependence, and complex instructions. Facilitators included self-discipline, personal responsibility, family and doctor support, and targeted health education. Themes of medication adherence, lack of shared decision-making, and suboptimal medication adherence emerged, highlighting the complexity of managing multiple medications among the study population. The study suggests enhancing participatory decision-making and support systems to improve adherence outcomes. | 4* |
| Peeters *et al.,* 2015. | Ghent, Belgium. | To explore the perspectives of Turkish migrants with type 2 diabetes on adherence to oral hypoglycaemic agents. | Qualitative study using in-depth interviews. | 21 participants, aged between 30-69 years. | All participants were of Turkish descent. | This study explores the perspectives of Turkish descent on adherence to oral hypoglycaemic agents. Key barriers to medication adherence included causal beliefs about stress and climate, adjusting medication around food intake or Ramadan fasting, and widespread use of herbal medicine. Facilitators included the religious interpretation of health and strong patient-provider relationships. The study highlights the complexity of medication adherence within this community, suggesting tailored approaches are necessary to improve outcomes. | 4* |
| Poon *et al.,* 2022. | Historically Black Community, Third Ward, Houston, USA | To understand perceptions about medication-related problems among older people from EMCs in a historically Black community. | Qualitative study using structured open-ended questions in focus group. | 10 practitioners participated in each meeting (including physicians, nurse practitioners, pharmacists, health educators, and social workers), 2-3 patients per meeting, with a total of 3 patients participating (patients were aged 65 years and above). | African American. | This study explores the perceptions of medication-related problems (MRP) among older people in a historically Black community. It highlighted the importance of patient-provider relationships, the impact of previous experiences and fears about medication side effects on medication adherence, and the need for improved medication management education and tools. The findings suggest significant roles of interpersonal interactions and personal experiences in managing complex medication regimens among minority elderly populations. | 4* |
| Robinson *et al.,*  2022 (A). | Primary care setting, United Kingdom | To explore barriers and facilitators to accessing medication reviews from the perspective of EMCs. | Qualitative study using semi-structured interviews | 20 participants, age range 26–75. | Participants' ethnicities included Asian or Asian British, Black African Caribbean or Black British, White, Other ethnic groups, and Mixed or multiple ethnic communities. | The study identified key barriers and facilitators affecting EMCs' access to medication reviews in the UK. Key findings reveal a general lack of awareness about medication review services and their benefits, highlighting the need for improved educational outreach and communication, possibly facilitated by community leaders and healthcare professionals. The study also emphasises significant language and communication barriers, suggesting that face-to-face interactions and the use of interpreters could enhance service accessibility. Additionally, it stresses the importance of healthcare providers understanding the cultural and religious contexts of their patients to offer culturally competent care and effectively address cultural stigmas associated with medication and health services. | The article included due to snowballing; 5*. |
| Robinson *et al.,* 2022 (B). | Primary, Secondary, and Tertiary Care, United Kingdom. | Explore barriers and facilitators affecting ethnic minority patient groups when accessing medicine review services from healthcare professionals' perspectives. | Qualitative study using semi-structured interviews | 18 practitioners (Ages range 20-69 years). | Practitioners' ethnicities included Indian, White, Arab, Black, Pakistani, and Bengali. | The study examined how healthcare professionals perceive barriers and facilitators to EMCs accessing medicine review services in the UK. Key findings include the need for cultural competency among practitioners to bridge the cultural divide and improve service accessibility. Additionally, the study highlighted the cultural stigma surrounding mental health, which often prevents these patients from seeking or adhering to treatments. Communication barriers were also significant, with recommendations for the use of multilingual resources and professional interpreters to enhance service delivery. The study underscores the importance of culturally sensitive practices and effective communication in improving healthcare access for EMCs. | The article included due to snowballing; 5*. |
| Secchi *et al.,* 2022. | United Kingdom (focus on the UK but not restricted to UK papers). | To review the literature on medication management in older ethnic minority communities in the UK. | A mixed-studies systematic review. | Older people aged 60 years and above, specific numbers not provided. | Asian, Black, and other EMCs. | This systematic review highlights the significant lack of research on medication management among older people from EMCs in the UK. Key challenges include cultural misbeliefs about medications, poor health literacy, and communication barriers that prevent effective medication management. Tailored educational interventions are suggested to improve outcomes in these communities. | 4* |
| Sultan *et al.,* 2023. | GP practices in East London, United Kingdom. | To understand how older Pakistani patients manage polypharmacy in their daily lives. | Narrative interview study. | 15 participants, aged over 50, all prescribed ten or more regular medications. | First-generation Pakistani migrants. | The study reveals those older Pakistani people in East London experience a complex interplay of cultural, family, and spiritual factors. Family involvement is central in managing multiple medications, reflecting deep-rooted cultural values. Spiritual beliefs also significantly influence how medications are perceived and adhered to. | 5* |
| Taskforce on Multiple Conditions,  2021. | Community-based settings in Stockwell (South London), Manningham and Toller (Bradford), Balsall Heath East and Sparkbrook (Birmingham), North Ormesby (Middlesbrough), United Kingdom. | To understand the experiences of individuals living with multiple long-term conditions and health inequity. | Ethnograpic research. | 52 participants (20 professionals and 32 individuals with multiple long-term conditions, ages not specified). | Diverse ethnicities and backgrounds including Black, Asian, communities. | This report explores systemic and structural barriers in healthcare, emphasising that personal and socioeconomic circumstances often limit individuals' choices in managing their health. It critiques the healthcare system for not providing equitable access or adequate support. Highlighting the need for healthcare to adapt to meet individuals' diverse needs. | Grey literature; 4*. |
| Thangsuk *et al.,* 2021. | Primary care clinic, Thailand | To determine if the relationship between herb use and high blood pressure in hypertensive patients is influenced by medication adherence. | Cross-sectional study. | 450 hypertensive patients aged 35 years and above. | Predominantly Thai. | This study examined how herbal medicine use affects blood pressure control in hypertensive patients, with a focus on medication adherence. Among 450 patients, 42% adhered well to their prescribed medications, while 18% used herbal remedies. The key finding was that high medication adherence strongly improved blood pressure control, whereas herbal use alone did not have a direct impact. The study highlights that adherence to prescribed medication is crucial for effective blood pressure management. Although this study was conducted in Thailand with a predominantly Thai population, the focus on herbal medicine use and its impact on medication adherence and blood pressure control is highly relevant. This is particularly important in understanding similar dynamics within EMCs in other countries who may also use traditional remedies. | 4* |
| Timsina *et al.,* 2022. | Family medicine clinic, United States. | To understand factors affecting diabetes medication adherence in Bhutanese refugees with type 2 diabetes. | Qualitative study using focus groups. | Total 4 practitioners (including nurses, physicians, and nurse practitioners), 13 patients with type 2 diabetes, averaging 58 years old (range 42-79) and 10 family members or caregivers. | Bhutanese refugees (Nepalese-speaking). | The study explored factors affecting medication adherence among Bhutanese refugees with type 2 diabetes. Key themes included the desire to engage in care but facing barriers, the value of family and community support, and the need for culturally contextual person-centered care. Specific recommendations were made to improve health literacy and tailored interventions to enhance medication adherence and health outcomes. | 4* |
| Upamali *et al.,* 2023. | Community and Diabetic Clinic, Peradeniya, Sri Lanka | To explore the perspectives of older people with uncontrolled type 2 diabetes on medication adherence. | Qualitative descriptive exploratory study. | 14 older people with uncontrolled type 2 diabetes mellitus, aged 60 years and above. | Participants were predominantly Sinhalese. | This study found that older individuals with uncontrolled type 2 diabetes face several barriers to medication adherence, including limited knowledge about their condition, negative attitudes toward medication, and difficulties related to aging. The role of family and healthcare support was also crucial, with some turning to alternative medicine. Although the study was conducted in Sri Lanka, its findings are relevant to South Asian communities in the UK, who face similar challenges in managing diabetes. | 4* |
| Yang *et al.,* 2021. | Family Practice Centre, Canada. | To explore the differing needs between Canadian- and foreign-born dementia patients and their caregivers. | Mixed methods: cross-sectional study followed by qualitative descriptive study. | 15 dementia patients, aged 61 years and above ,and 15 caregivers. | Canadian-born and foreign-born patients and caregivers. | The study revealed that foreign-born people living with dementia and their caregivers have more extensive needs and experience higher stress compared to their Canadian-born counterparts, pointing to the significant impact of cultural background on dementia care needs. Despite these differences in needs and stress levels, both groups reported similar experiences with the healthcare system, suggesting that cultural diversity influences specific care requirements but does not alter the general perception of the system's effectiveness. This underscores the importance of culturally sensitive approaches in dementia care to better accommodate the diverse needs of the population. | 4* |
| Yoon *et al.,* 2023. | Community hospital, Singapore. | To explore cultural perspectives on factors influencing medication adherence among patients with chronic diseases in a multi-ethnic Asian setting. | Qualitative study with in-depth interviews. | 25 participants, with ages ranging from 25 to 78 years. | Participants were predominantly Chinese, followed by Malay, Indian. | The study looked at how cultural and systemic factors affect medication adherence among South Asians, especially those of Indian descent. It identified challenges like side effects, forgetfulness, language barriers, and cultural views on modern medicine. To improve adherence, the study recommends better communication between doctors and patients, more coordinated care, and education tailored to cultural needs. Although the study was conducted in Singapore, the barriers identified—such as cultural beliefs about modern medicine, language issues, and the impact of side effects—are also relevant to South Asian communities in the UK. | 5* |
